# Long-term outcomes of left atrial appendage closure with or without concomitant pulmonary vein isolation:a propensity score matching analysis based on CLACBAC study

**DOI:** 10.1186/s12872-024-03725-1

**Published:** 2024-02-03

**Authors:** Xiang Li, Shiyu Feng, Zhongyuan Ren, Jiayu Wu, Lili Zhou, Haotian Yang, Yixing Zheng, Weilun Meng, Jun Zhang, Yang Su, Yan Jiang, Jun Xu, Hui Sun, Yawei Xu, Dongdong Zhao, Xiaobing Yin

**Affiliations:** 1 Tongji University School of Medicine, Shanghai, 200092 China; 2 Heart Centre, Shanghai Tenth People’s Hospital, School of Medicine, Tongji University, Shanghai, 200092 China; 3 Department of Nursing, Shanghai Tenth People’s Hospital, Tongji University School of Medicine, Shanghai, 200092 China; 4 School of Clinical Medicine, Shanghai University of Medicine and Health Sciences, Shanghai, 201318 China; 5 School of Medicine, Anhui University of Science and Technology, Anhui province, Huainan, China; 6 Department of Cardiology, Shanghai Tenth People’s Hospital Chongming Branch, Shanghai, 202157 China

**Keywords:** Atrial fibrillation, Pulmonary vein ablation, Left atrial appendage closure, Propensity matching study, Long-term benefits

## Abstract

**Background:**

The combined procedure of left atrial appendage closure (LAAC) with concomitant pulmonary vein isolation (PVI) has demonstrated its efficacy and safety. However, there is still a lack of comparative investigations regarding the long-term benefits of the combined procedure when compared to LAAC alone. Our study aims to assess the long-term outcomes of combined procedure of LAAC with concomitant PVI in comparison with a propensity matched LAAC alone group.

**Methods:**

Propensity score matching (PSM) was employed to rectify covariate imbalances, resulting in the inclusion of 153 comparable patients from the initial cohort of 333 non-valvular atrial fibrillation (AF) patients. Clinical outcomes, encompassing thrombotic events, major cardiocerebrovascular adverse events (MACCE), re-hospitalization due to cardiovascular disease (CVD), and atrial tachycardia (AT), were juxtaposed between the two groups. Bleeding events and peri-device complications, such as residual flow, device-related thrombus, and device replacement, were also compared. Additionally, a patients group underwent PVI alone was included for comparing AF recurrence rates between the PVI alone group and the combined group.

**Results:**

Following PSM, 153 patients (mean age 70.3 ± 8.9, 62.7% men) were included, with 102 undergoing the combined procedure and 51 undergoing LAAC alone. No significant differences were found in baseline characteristics between the two groups. The mean follow-up time was 37.6 ± 7.9 months, and two patients were lost to follow-up in the combined procedure group. Thrombotic events were observed in 4 (7.8%) patients in the LAAC alone group and 4 (4.0%) in the combined group (Log-rank *p* = 0.301). The proportion of patients experiencing MACCE, re-hospitalization due to CVD, and AT between the two groups was comparable, as were bleeding events and peri-device complications. Among patients from the combined procedure group without AF recurrence, a significant difference was noted in prior-procedure left ventricular ejection fraction (LVEF) and LVEF at the 12th month after the procedure (57.2% ± 7.1% vs. 60.5% ± 6.5%, *p* = 0.002).

**Conclusion:**

The concomitant PVI and LAAC procedure did not increase procedure-related complications, nor did it confer significant benefits in preventing thrombotic events or reducing other cardiovascular events. However, the combined procedure improved heart function, suggesting potential long-term benefits.

**Supplementary Information:**

The online version contains supplementary material available at 10.1186/s12872-024-03725-1.

## Background

The global management of atrial fibrillation (AF) constitutes a substantial social and economic burden. From one vantage point, stroke and thromboembolic events stand as prominent contributors to mortality and disability in patients with non-valvular AF [1].

In non-valvular AF patients, the left atrial appendage (LAA) chamber manifests a procoagulant microenvironment [2], with over 90% of clots originating from the left atrium (LA) believed to derive from the LAA [3]. Recognizing the LAA as a primary source of thrombus in non-valvular AF patients [4], the exclusion of the left atrial appendage has emerged as a promising alternative to mitigate the occurrence of thromboembolic events [5]. Meanwhile, an alternative perspective in AF management emphasizes rhythm control [6].

Among the various strategies, catheter-based pulmonary vein isolation (PVI) is deemed a satisfactory intervention for paroxysmal and early persistent AF patients [7, 8]. The CABANA trial results have underscored that catheter ablation surpasses antiarrhythmic drugs in efficacy for rhythm control [9].

More recently, the integration of catheter ablation with left atrial appendage closure (LAAC) in a singular procedure has demonstrated both feasibility and safety, becoming a widely adopted strategy in clinical practice. Theoretically, the amalgamation of LAAC with catheter ablation is poised to confer additional benefits to atrial fibrillation (AF) patients. This combined approach not only achieves rhythm control but also mitigates the risk of thromboembolic events. Despite some published studies affirming the efficacy and safety of the combined LAAC + PVI procedure [9–11], the enduring advantages of concomitant pulmonary vein isolation (PVI) with LAAC, as opposed to LAAC alone, in non-valvular AF patients remain unvalidated. This lack of validation is attributed to the limited study population and the absence of controlled designs in existing research. Additionally, concerns persist regarding potential complications, such as oedema induced by ablation impacting LAAC device implantation and causing peri-device leakage due to underestimation of the left atrial appendage (LAA) diameter and device size [12]. Furthermore, LAAC may potentially reduce atrial volume, influencing left atrial function and possibly compromising the prognosis of PVI [13].

Therefore, our objective is to compare the combined procedure of LAAC + PVI with LAAC alone, utilizing propensity score-matched data collected from our centre. Through this analysis, we aim to investigate the safety and long-term outcomes associated with the concomitant LAAC + PVI procedure and provide valuable insights for clinical practice.

## Method

### Study population

This retrospective cohort study utilized data from the registered “Combining Left Atrial Appendage Closure with Cryoballoon Ablation in the Chinese Population” trial (CLACBAC, registration number NCT04185142). The study encompassed 333 consecutive non-valvular atrial fibrillation (AF) patients who underwent either left atrial appendage closure alone (*n* = 134) or a combination of left atrial appendage closure and pulmonary vein isolation (LAAC + PVI) (*n* = 199) at Shanghai Tenth People’s Hospital between May 2017 and November 2019. Inclusion criteria comprised [1] patients diagnosed with AF, whether persistent or paroxysmal [2]; patients who underwent percutaneous LAAC alone or LAAC + PVI procedures [3]; CHA2DS2-VASc score ≥ 2 or HAS-BLED score ≥ 3. Exclusion criteria included [1] valvular heart disease [2]; patients with arrhythmias other than AF [3]; patients who underwent interventions in the intracardiac cavity other than LAAC and PVI [4]; patients in whom occluder implantation failed for any reason. Additionally, a cohort of patients who underwent PVI alone was included for comparing AF recurrence rates between the PVI alone group and the combined group. Propensity score matching between these two groups was performed to balance baseline differences. The study received approval from the Medical Research Ethics Committee of Shanghai Tenth People’s Hospital, and all procedures were conducted following thorough pre-procedural evaluation upon hospital admission.

### Procedure access

Local anaesthesia in the groin region was achieved using lidocaine, followed by a right femoral vein puncture, serving as the access point for subsequent ablation and left atrial appendage closure (LAAC). Utilizing digital subtraction angiography (DSA), a Swartz Sheath was advanced into the right atrium, followed by the creation of transseptal access through septum puncture.

### Catheter ablation procedure

Pulmonary vein isolation was uniformly accomplished in all patients through cryoballoon ablation, employing a 23 mm or 28 mm first or second-generation cryoballoon. A standard freeze time of 180 seconds, coupled with a time-to-isolation adjustment strategy, was applied for each pulmonary vein isolation. Verification of pulmonary vein isolation was conducted using the Achieve catheter (Achieve, Medtronic, Minneapolis, MN).

### LAAC procedure

Following cryoballoon ablation, LAAC was promptly conducted. A comprehensive description of the LAAC procedure has been previously provided [10]. Both plug occluders (WATCHMAN; Boston Scientific, MA, USA) and pacifier occluders (LAmbre device, Lifetech Scientific, Shenzhen, China) were utilized. The occluder device was delivered through a 14 F delivery sheath under the guidance of transoesophageal echocardiography (TOE) and fluoroscopy.

For plug occluders, the size was selected to be 4-6 mm larger than the measured diameter of the left atrial appendage (LAA) ostium, ensuring an adequate compression ratio and stable positioning. In the case of pacifier occluders, the size of the outer plate was maintained 2–3 mm larger than the measured diameter of the LAA orifice, ensuring complete sealing.

Prior to device release, the PASS (position, anchor, size, seal) principle for the WATCHMAN device and COST (circumflex artery, open, sealing, tug test) principle for the LAmbre device were meticulously fulfilled and confirmed through TOE and angiography. Evaluation of positioning, compression ratio, residual flow, and procedure-related complications occurred through TOE and LA angiography upon device release.

### Periprocedural complications

Periprocedural complications encompassed pericardial effusion (with and without drainage), transoesophageal echocardiography (TOE) intolerance, phrenic nerve palsy, vasovagal episode, and femoral access site problems. Femoral access site problems entailed complications arising from venipuncture or inadvertent injury to the adjacent artery, encompassing hematoma and bleeding necessitating transfusion (major bleeding).

Post-procedural, peri-device leakage (PDL) was identified via TOE. PDL with a size < 1 mm was categorized as mild leaks, those within the range of 1 mm to 5 mm were considered moderate leaks, and those exceeding 5 mm were classified as severe leaks. For the purposes of this study, only moderate leaks and severe leaks were taken into consideration.

### Follow-up

Patients were mandated to undergo outpatient follow-ups at the 1st, 3rd, 6th, and 12th months post-procedure, followed by at least annual follow-ups thereafter. Clinical outcomes were monitored through outpatient visits and trans-telephonic follow-ups every 3 months post-procedure.

Transoesophageal echocardiography (TOE) was conducted at the 3rd and 12th months to confirm device-related complications such as device displacement, device-related thrombus, residual flow, and pericardial effusion. Trans Thoracic Echocardiography (TTE) was employed to assess changes in patients’ heart function, measuring left atrial diameter (LAD) and left ventricular ejection fractions (LVEF) before the procedure and at the 12th month post-procedure.

### Clinical outcomes

Clinical outcomes comprised thrombotic events (TE), encompassing ischemic stroke, transient ischemic attack, and peripheral arterial embolism. Major adverse cardiocerebrovascular events (MACCE), re-hospitalization due to cardiovascular disease (CVD), and re-hospitalization due to atrial tachycardia (AT) were also assessed. Atrial tachycardia (AT) was defined as recorded atrial fibrillation (AF), atrial flutter (AFL), and atrial tachycardia lasting longer than 30 seconds post the 3rd month following the procedure.

### Antithrombotic therapy after the procedure

All patients were generally administered antiarrhythmic drugs during the blank period. As part of anticoagulation strategy, warfarin or new oral anticoagulants (NOACs, dabigatran or rivaroxaban) were recommended to all patients for a duration of 2–3 months to prevent device-related thrombosis. Transoesophageal echocardiography (TOE) was conducted at the 3rd month to assess device position, identify new peri-device residual flow, and confirm the absence of device-related thrombosis. Upon confirmation of satisfactory occlusion by TOE, oral anticoagulants (OACs) were discontinued, and double antiplatelet drugs were administered until the 6th month. Subsequently, a single antiplatelet drug (aspirin or clopidogrel) was prescribed as a lifelong therapy.

### Statistical analysis

All data were analysed utilizing SPSS 26.0 (IBM, Armonk, NY, USA). Continuous variables assuming a normal distribution were compared using Student’s t-test, and results were presented as mean values ± standard deviation (SD). In cases where continuous variables did not assume normal distribution, the Mann-Whitney U test was employed, and results were presented as median with interquartile range.

Categorical variables were described as numbers with percentages, and the comparison between two groups was conducted using the Chi-square test and Fisher’s exact test.

For survival analysis, Kaplan-Meier curves and Log-rank tests were employed to estimate the difference in the freedom of endpoint events between the two groups. A 1:2 propensity score matching (PSM) was implemented, fitting a multivariate logistic regression model to eliminate co-variate imbalances in baseline characteristics between groups. The matched co-variates included AF type, left atrial diameter (LAD) before the procedure, sex, and age.

## Results

### Patients baseline characteristics

Initially, among the 333 patients enrolled, 134 underwent the LAAC alone procedure, while 199 received the combined procedure. However, there were significant differences in some crucial baseline characteristics (as detailed in Table 1). Subsequently, a 1:2 propensity score matching (PSM, LAAC alone group vs combined procedure group) was executed to rectify the co-variate imbalance in baseline variables. Following PSM, a total of 153 patients (mean age 70.3 ± 8.9, 62.7% men) were included, with 51 undergoing LAAC alone and 102 undergoing the combined procedure. There were no significant differences in age (69.7 ± 1.1 vs. 70.6 ± 1.0), gender, CHA2DS2-VASc score, and HAS-BLED score. Both groups exhibited similar heart and renal function, as indicated by proBNP and eGFR. Regarding prior disease history, coronary artery disease was defined as a lumen narrowing of more than 50% in any coronary artery on previous coronary angiography. No significant differences were observed in prior disease and medical treatment history between the two groups. The previous use of class I/III antiarrhythmic drugs was comparable between the two groups. Further details of baseline characteristics are presented in Table 1.

**Table 1:** Baseline characteristics of LAAC alone group and combined procedure group

Baseline Characteristics	Before PSM		*P* value	After PSM		*P* value
	LAAC alone (*n* = 134)	Combined procedure (*n* = 199)		LAAC alone (*n* = 51)	Combined procedure (*n* = 102)	
Age, years	73.0[67.0,79.0]	69.0[63.0,77.0]	0.001*	69.7 ± 1.1	70.6 ± 1.0	0.532
Men, n (%)	85(63.4)	117(58.8)	0.396	31(60.8)	65(64.4)	0.723
BMI, kg/m^2^	25.4 ± 3.1	25.7 ± 3.8	0.535	25.3 ± 0.4	25.8 ± 0.4	0.456
Persistent atrial fibrillation (%)	108(81.8)	110(55.3)	<0.001*	21(41.2)	39(38.6)	0.725
CHA2DS2-VASC score	4.0[3.0,5.0]	3.0[2.0,5.0]	0.004*	4.0[3.0,5.0]	3.0[2.0,5.0]	0.266
HAS-BLED score	3.0[2.0,3.0]	2.0[2.0,3.0]	0.008*	3.0[2.0,3.0]	2.0[2.0,3.0]	0.083
pro BNP, pg/ml	948.2[518.71622.0]	680.9[246.21362.0]	0.007*	866.5[496.31550.0]	646.2[302.81603.5]	0.249
eGFR, ml/min/1.73m^2^	84.4[71.0,101.7]	85.4[71.4,99.4]	0.911	93.1[69.2112.3]	88.0[76.1102.6]	0.341
Previous illness						
Previous AF ablation, n (%)	13(9.7)	11(5.5)	0.149	2 (15.7)	3 (2.9)	0.748
Pacemaker, n (%)	9(6.9)	14(7.2)	0.930	4(7.8)	9(8.9)	1.000
Stroke, n (%)	53(39.6)	54(27.1)	0.017*	23(45.1)	44(43.1)	0.818
Cerebral infraction, n (%)	52(38.8)	52(26.1)	0.014*	22(43.1)	43(42.2)	0.908
Intracranial haemorrhage, n (%)	5(3.7)	5(2.5)	0.755	4(7.8)	2(2.0)	0.077
Heart failure, n (%)	28(20.9)	38(19.1)	0.686	6(11.8)	18(17.7)	0.346
COPD, n (%)	2(1.5)	7(3.5)	0.472	0(0.0)	2(2.0)	–
Hypertension, n (%)	104(77.6)	145(72.9)	0.328	37(72.6)	72(70.6)	0.801
Diabetes mellitus, n (%)	37(27.6)	52(26.1)	0.765	14(27.5)	24(23.5)	0.597
Perivascular disease, n (%)	7(5.2)	11(5.5)	0.904	3(6.0)	2(2.0)	0.334
Coronary heart disease, n (%)	40(29.9)	64(32.2)	0.656	15(29.4)	32(31.4)	0.804
Previous medication						
Class I/III AADs, n (%)	10(7.5)	52(26.1)	<0.001*	15(29.4)	29(28.4)	0.951
Oral anti-coagulants, n (%)	56(41.8)	63(31.7)	0.059	22(43.1)	33(32.4)	0.190
NOAC, n (%)	35(26.1)	39(19.6)	0.160	14(27.5)	21(20.6)	0.341
Warfarin, n (%)	21(15.7)	21(12.1)	0.345	8(15.7)	12(11.8)	0.498
Anti-platelet drugs, n (%)	39(29.1)	62(31.2)	0.690	16(31.4)	29(28.4)	0.707

*eGFR* estimated glomerular filtration rate, *COPD* chronic obstructive pulmonary disease, *AADs* anti-arrhythmic drugs, *AF* atrial fibrillation, *NOAC* new oral anti-coagulants peri-procedural complications

### Procedure access

Four patients were found to have PFO/ASD screened by preprocedural TOE examination. In one of these patients, access to left atrium via PFO were used. However, since trans-PFO access made co-axiality of the catheter sheath and the left atrial appendage unsatisfactory because of the cranial position of PFO, trans septum access by septal puncture was subsequently performed in this patient to enter the left atrium. In the rest of patients with no PFO/ASD, trans-septal access was achieved after septal puncture.

### Procedural details

All patients underwent LAAC alone and combined procedure successfully, with only 9 in total who experienced peri-procedural complications.

The incidence rate of peri-procedural complications, which was defined as the sum of procedure-related complications that occur to the patient in the prior-procedural examination, during the procedure, and 7 days after the procedure between LAAC alone group and combined procedure group, were comparable (5.9% vs 5.9%, *p* = 1.000). 1 patient in LAAC alone group and 4 in combined procedure group were found pericardial effusion (2.0% vs 3.9%, *p* = 0.663). Femoral access site problems were found in one patient in LAAC alone group and one in combined procedure group (2.0% vs 1.0%, *p* = 0.998), with one hematoma in LAAC alone group and one major bleeding in combined group. Groin hematoma was caused by unintentional injury of femoral artery when performing femoral vein puncture, and there were no intentional arterial punctures in the procedure process. Besides, there were only one patient experienced TOE intolerance and one observed phrenic nerve palsy, and both of them were from combined procedure group, while one patient in LAAC alone group were observed vagal reflex.

Regarding the type of LAAC device, 135 patients (88.2%) received the plug occluder (WATCHMAN device), while the remaining 11.8% opted for the pacifier occluder (LAmbre device). The distribution of patients adopting either the plug occluder or pacifier occluder in the two groups was comparable.

In terms of device resizing, 9.8% of patients undergoing LAAC alone experienced this, while the proportion in the combined procedure group was 5.9% (*p* = 0.376). All patients achieved satisfactory LAAC sealing. Four patients were observed to have peri-device leakages (≥1 mm) by TOE/fluoroscopy after the LAAC procedure, with 3 in the LAAC alone group and 1 in the combined procedure group (3.1% vs. 2.0%, *p* = 0.706). Additional details are provided in Table 2.

**Table 2:** Peri-procedural examination and details in the procedure

	LAAC alone (*n* = 51)	Combined procedure (*n* = 102)	*P* value
**Peri-procedural examination**			
Left atrial diameter, mm	44.7 ± 0.7	43.5 ± 0.5	0.159
LVEF, %	60 [56,62]	60 [56,60]	0.142
Previous pericardial effusion, n (%)	5 (9.8)	13 (12.8)	0.595
Mean LAA measurement			
LAA mean open diameter, mm	20.2 ± 0.6	20.5 ± 0.4	0.681
LAA mean depth, mm	21.5 ± 0.7	20.9 ± 0.4	0.463
**Procedure related data**			
Device related parameter			
Plug occluder, n (%)	45 (88.2)	90 (88.3)	1.000
Plug size, mm	27.0 [24.0,33.0]	27.0 [24.0,30.0]	0.495
Pacifier occluder, n (%)	6 (11.8)	12 (11.8)	1.000
Device size, mm	29.5 [28.0,34.0]	32.5 [29.5,36.0]	0.142
Plug size, mm	25.0 [23.25,28]	26 [24.3,29.8]	0.374
Compression ratio, %	19.0 [16.7,22.6]	20.5 [18.0,25.0]	0.064
Deploy times, n ± SD	1.3 ± 0.1	1.4 ± 0.1	0.051
Change device size (≥1), n (%)	5 (9.8)	6 (5.9)	0.376
Peri device leakage			
Leakage ≥1 mm, n (%)	1/49 (2.0)	3/96 (3.1)	0.706
Leakage ≥5 mm, n (%)	0	1	–
**Periprocedural complications**	3 (5.9)	6 (5.9)	1.000
Pericardial effusion, n (%)	1 (2.0)	4 (3.9)	0.663
With drainage, n (%)	1 (2.0)	2 (2.0)	0.998
Without drainage, n (%)	0	2 (2.0)	–
TOE intolerance, n (%)	0	1 (1.0)	–
Phrenic nerve palsy, n (%)	0	1 (1.0)	–
Vasovagal episode, n (%)	1 (2.0)	0	–
Femoral access site problems, n (%)	1 (2.0)	1 (1.0)	0.998
Groin hematomas, n (%)	1 (2.0)	0	–
Major bleeding, n (%)	0	1 (1.0)	–

*LVEF* left atrial ejection fraction, *LAA* left atrial appendage, *TOE* Transoesophageal echocardiography

### Clinical outcomes

The average length of hospital stay was comparable between the LAAC alone group and the combined group (4.1 ± 1.1 days vs. 4.3 ± 1.4 days, *p* = 0.897), with no outpatient procedures included. The mean follow-up time was 37.3 ± 10.4 months in the LAAC group and 37.7 ± 6.5 months in the combined procedure group (*p* = 0.763), with 2 patients in the combined procedure group lost to follow-up. Five re-ablations were performed in patients with recurrence of atrial fibrillation after a combined procedure, while no ablation was performed in patients after isolated left atrial appendage closure.

During the follow-up period, a total of 8 (5.3%) patients experienced thrombotic events, with 4 in each group. Among these, 5 patients had ischemic strokes, with 2 in the LAAC alone group and 3 in the LAAC + PVI group (3.9% vs. 3.0%, log-rank *p* = 0.736), and 3 patients had peripheral arterial embolisms, with 2 in the LAAC alone group and 1 in the combined procedure group (3.9% vs. 1.0%, log-rank *p* = 0.210).

**Fig. 1:**
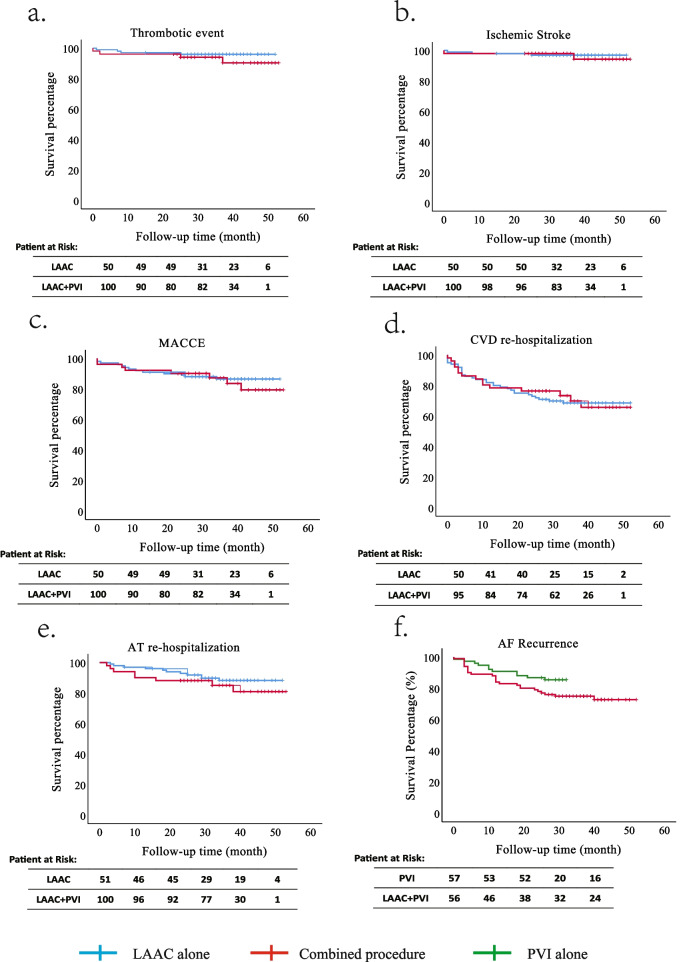
Survival curves of clinical outcomes. **a** Survival curve for thrombotic events; **b,** Survival curve for ischemic stroke; **c,** Survival curve for MACCE; **d,** Survival curve for CVD re-hospitalization; **e,** Survival curve for AT re-hospitalization; **f,** Survival curve for AF recurrence of combined procedure group and PVI only group. No significant difference was observed among groups. MACCE, major adverse cerebrocardiovascular events; CVD, cardiovascular disease, and AT, atrial tachycardia

Regarding other clinical outcome events, the incidence rates of major adverse cardiocerebrovascular events (MACCE), re-hospitalization due to cardiovascular disease (CVD), and re-hospitalization due to atrial tachycardia (AT) were comparable between the two groups. No patient deaths occurred during the follow-up period. Regarding haemorrhagic events, 1 patient in the LAAC alone group and 2 patients in the combined procedure group experienced major bleeding (2.0% vs. 2.0%, *p* = 0.989). Detailed information on clinical outcomes is listed in Table 3, and Kaplan-Meier curves of clinical outcomes are presented in Fig. 1.

**Table 3:** Clinical outcomes and complications in LAAC alone group and combined procedure group during follow-up

	LAAC alone (*n* = 51)	Combined procedure (*n* = 100)	Log-rank *p* value
**Clinical outcomes**			
Thrombotic events, n (%)	4 (7.8)	4 (4.0)	0.301
Ischemic stroke and TIA, n (%)	2 (3.9)	3 (3.0)	0.736
PAE, n (%)	2 (3.9)	1 (1.0)	0.210
MACCE, n (%)	8 (15.7)	13 (13.0)	0.653
CVD re-hospitalization, n (%)	15 (29.4)	31 (31.0)	0.951
AT re-hospitalization, n (%)	8 (15.7)	11 (11.0)	0.313
Major haemorrhagic event, n (%)	1 (2.0%)	2 (2.0)	0.989
**Complications**			
Mild leakage (≥1 mm), n (%)	11 (21.6)	18 (18.0)	0.666
Device-related thrombus, n (%)	0	0	–
Device displacement, n (%)	0	0	–
Major bleeding, n (%)	1 (2.0)	2 (2.0)	0.989
Minor bleeding, n (%)	0	1 (1.0)	–

*TIA* Transient ischemic attack, *PAE* Peripheral Arterial Embolism, *MACCE* Major Adverse Cardiocerebrovascular Events.

Thirty-one patients in the LAAC alone group and 61 in the combined procedure group underwent both prior-procedural Transthoracic Echocardiography (TTE) examination and repeat TTE examination at the 12th month after the procedure, with measurements of Left Atrial Diameter (LAD) and Left Ventricular Ejection Fraction (LVEF). Considering the potential impact of AF recurrence on LAD and LVEF, the combined procedure group was divided into an AF recurrence group (*n* = 8) and a non-AF recurrence group (*n* = 53) based on the presence or absence of AF recurrence before the 12th month of follow-up. Between-group and within-group comparisons were conducted.

**Fig. 2:**
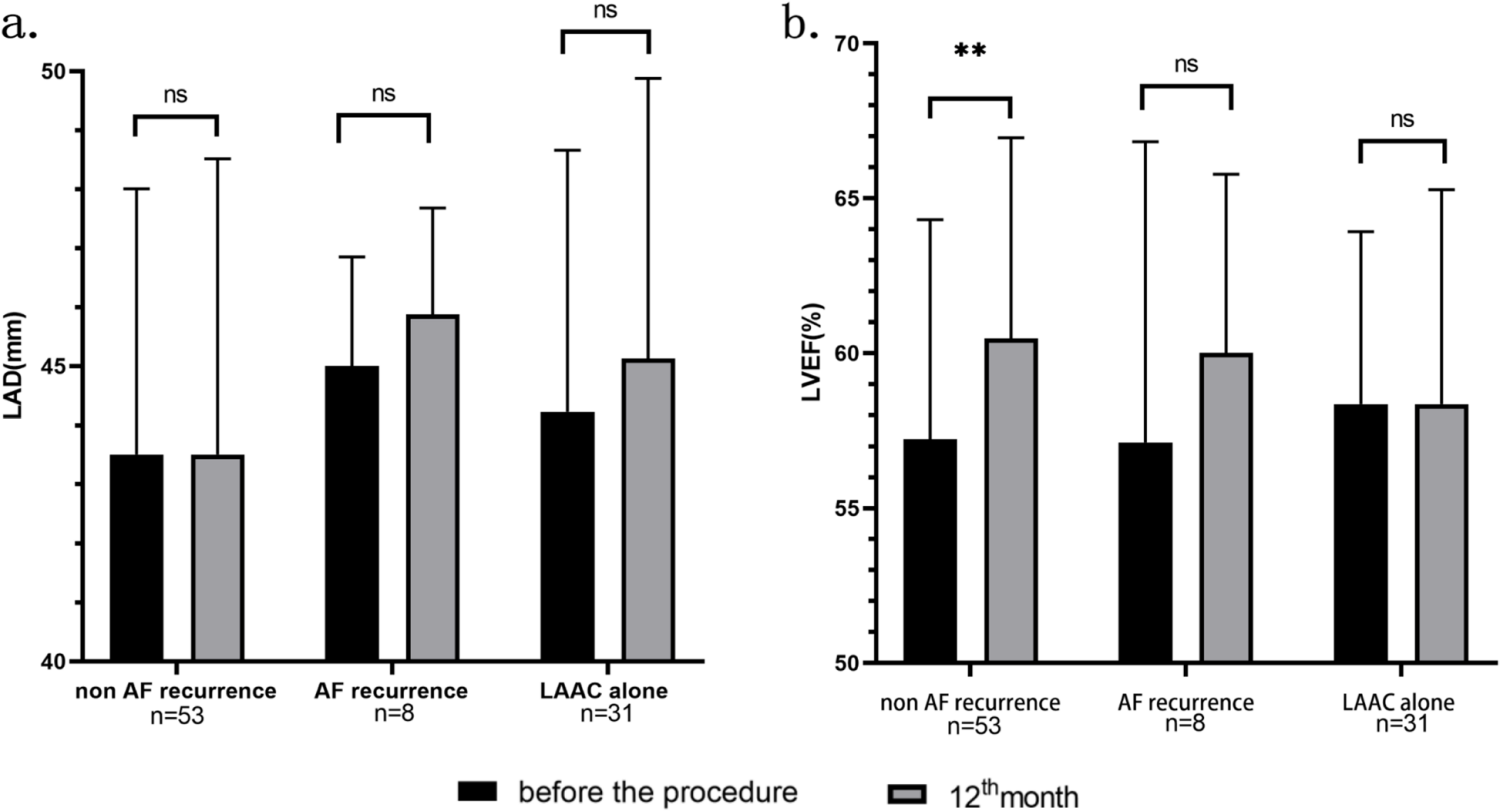
Comparison of LAD and LVEF among combined procedure groups with/without AF recurrence with LAAC group. **a** comparison of LAD; **b,** comparison of LVEF. The average LVEF for combined procedure group without AF recurrence showed a significant improvement at 12th month TTE examination

No significant differences in LAD and LVEF were found within the three groups in the prior-procedure TTE examination. The 12th-month TTE examination revealed that patients in the AF recurrence group had a significantly larger average LAD than those in the non-AF recurrence group (45.9 mm ± 1.8 mm vs. 43.5 mm ± 5.0 mm, *p* = 0.046). Furthermore, compared to the prior-procedure LVEF, although all three groups showed an increase in average LVEF at the 12th-month TTE examination, only the non-AF recurrence group exhibited a significant increase in average LVEF (57.2% ± 7.1% vs. 60.5% ± 6.5%, *p* = 0.002). Related details are shown in Fig. 2.

### Pei-procedural complications

All patients underwent Transoesophageal Echocardiography (TOE) examinations at the 3rd month, 12th month, and annually after the procedure. TOE at the 12th month revealed mild residual leakage in 29 patients, with 11 (21.6%) in the LAAC alone group and 18 (18.0%) in the LAAC + PVI group. Among these cases, a maximum of 3 mm residual flow was detected in one patient, and no residual flow exceeding 5 mm was observed. No device-related thrombosis, displacement of the Left Atrial Appendage Closure (LAAC) device, or pericardial effusion were observed in either group.

### Antithrombotic strategy after the procedure

Although all patients were advised anticoagulation therapy during the first 3 months after the procedure, 20 (13.2%) patients did not take Oral Anticoagulants (OACs) during this period due to contraindications. Among these patients, 5 (5.0%) in the combined procedure group and 2 (3.9%) in the LAAC alone group received no antithrombotic therapy due to their high bleeding risk indicated by the HAS-BLED score. For the remaining 13 patients, 8 (8.0%) in the combined procedure group and 5 (9.8%) in the LAAC alone group were prescribed single antiplatelet therapy. No Device-Related Thrombosis (DRT) was observed in these 20 patients during the follow-up.

After the first 3 months of follow-up, 44 patients in the LAAC alone group and 85 in the combined procedure group were recommended to shift from OACs to Single Antiplatelet Therapy (SAPT) or Dual Antiplatelet Therapy (DAPT). Two in the LAAC alone group and 10 in the combined group continued on Non-Vitamin K Oral Anticoagulants (NOACs) due to peri-device thrombosis confirmed by Transoesophageal Echocardiography (TOE). By the end of the follow-up, 11 (7.3%) patients were on DAPT, with 3 (5.9%) in the LAAC alone group and 7 (7.0%) in the combined procedure group, and 125 (82.8%) patients were on SAPT, with 36 (70.6%) in the LAAC alone group and 81 (81.0%) in the combined procedure group.

**Fig. 3:**
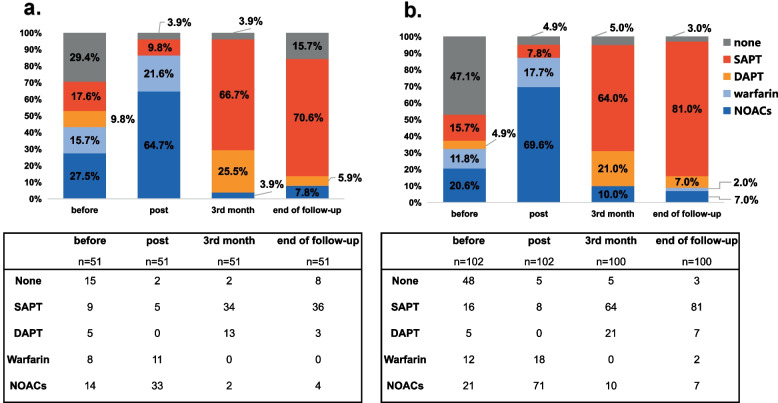
Shift of anti-thrombotic therapy before procedure, post procedure and by the 3rd and 12th month since the procedure**.** DAPT, double anti-platelet therapy; SAPT, single anti-platelet therapy and NOACs, new oral anti coagulants

It is noteworthy that 4 in the LAAC alone group and 9 in the combined procedure group shifted back to OACs from antiplatelets due to thrombotic events during this period. Additionally, 8 in the LAAC alone group and 3 in the combined procedure group discontinued antithrombotic therapy due to bleeding events. Overall, the withdrawal of OACs in patients during follow-up showed a downward trend, as displayed in Fig. 3.

## Discussion

Since the initial report by Swaans et al. on the feasibility of combining Pulmonary Vein Isolation (PVI) with Left Atrial Appendage Closure (LAAC) in a single procedure [11], this dual approach has been considered a promising strategy for non-valvular Atrial Fibrillation (AF) patients at high risk of stroke and bleeding. Despite this, existing evidence does not strongly support the assertion that the combined procedure offers superior long-term clinical outcomes compared to isolated LAAC. In our study comparing the combined procedure of LAAC + PVI with LAAC alone, several key findings emerged: (i) Periprocedural Safety: PVI did not increase periprocedural complications and device-related adverse events associated with LAAC. (ii) Long-term Efficacy: The combined procedure of LAAC + PVI demonstrated similar efficacy to LAAC alone in terms of long-term AF-related clinical outcomes in patients with non-valvular AF. (iii) Heart Function Improvement: The combined procedure of LAAC + PVI led to a more pronounced improvement in heart function compared to LAAC alone.

These findings contribute valuable insights to the ongoing discourse on the optimal management strategy for non-valvular AF patients, shedding light on both the safety and efficacy aspects of the combined LAAC + PVI approach.

### Feasibility and safety of combined procedure

The LAAC procedure has emerged as a viable alternative to Oral Anticoagulants (OACs) for preventing ischemic stroke in Atrial Fibrillation (AF) patients, offering the advantage of reduced bleeding risk compared to long-term OAC use. Simultaneously, rhythm control, encompassing both pharmacological and ablative approaches, plays a crucial role in improving patient symptoms. Results from the randomized CABANA trial have underscored the superiority of catheter ablation (CA) over antiarrhythmic drugs in AF patients [9, 14]. Therefore, the prospect of combining LAAC and CA in a single procedure, sharing a common access point, appears to be an appealing strategy for symptomatic AF patients, addressing both stroke prevention and rhythm control.

Previous studies have demonstrated the feasibility and safety of undergoing a combined procedure of LAAC and Pulmonary Vein Isolation (PVI) in a single session for AF patients. The initial study, reporting on the feasibility and safety of the combined procedure, revealed a complete occlusion rate of 77.0%, which increased to 93.0% at the Transoesophageal Echocardiography (TOE) examination conducted 6 months later [11]. A recent multi-centre study from China reported an average occlusion rate of 94.3% in patients undergoing the combined procedure [15]. Notably, even when using two different types of devices (WATCHMAN and ACP), the complete occlusion rates remained comparable (92.8% in the WATCHMAN group and 97.4% in the ACP group).

Data from the CLACBAC study, conducted at our centre, demonstrated an overall success rate of 97.3% for the combined procedure, with 76 patients receiving three different types of LAAC devices (WATCHMAN, Lafort, and Lacbes, respectively) [10]. This evidence collectively establishes the feasibility of the combined procedure of LAAC and PVI, attesting to its safety and success rates.

While combined procedures inherently increase the complication rate compared to individual procedures (catheter ablation or LAAC), the amalgamation of two procedures in a single session appears more patient-friendly than performing them separately. A Propensity Score Matching (PSM) study by Mo et al. comparing single and combined procedures reported a complication rate of 3.9% in the combined procedure group, slightly higher than the rates of 2.6% in both the catheter ablation (CA) alone and LAAC alone groups [16]. Notably, Wintgens et al. demonstrated a comparable complication rate of 4% in their study discussing a staged procedure of CA after LAAC [17]. In our study, we reported a slightly higher complication rate of 5.9% in both the combined procedure and LAAC alone groups, with no significant difference between the two. This discrepancy may be attributed to the relatively small cohort size. Moreover, compared to performing procedures separately, combining two procedures may help avoid complications in common procedural aspects such as groin hematoma and bleeding caused by femoral vein puncture.

Major complications primarily stem from femoral vascular access-site issues, transeptal puncture-related access-site problems like pericardial effusion (PE), device-related thrombosis (DRT), and peripheral device leakage (PDL)/residual leakage. Among these, femoral access-site problems and PE are linked to operators’ experience. Previous studies comparing combined procedures with LAAC alone have shown a low incidence of femoral access site problems and PE, with no significant difference between the two groups [16]. In our study, the incidence of femoral access site problems and PE in the combined group and LAAC alone group were comparable and lower than those reported in previous studies [16, 18]. This suggests that operators’ experience, rather than the procedure itself, influences the incidence of complications.

For patients with previously identified patent foramen oval (PFO) or atrial septal defect (ASD), performing left atrial appendage closure (LAAC) via PFO/ASD can be considered as an alternative to reach the left atrium and concurrently avoid access site problems associated with transeptal puncture. A single-centre cohort study demonstrated that, when compared to LAAC with transeptal puncture access, LAAC via trans-PFO/ASD access achieved comparable results in terms of procedure success rate, procedural-related complications rate, and the incidence rate of cardiovascular adverse events during follow-up [19]. It is noteworthy, however, that the co-axiality of the catheter sheath and the left atrial appendage may be affected by the relatively cranial position of the PFO, potentially resulting in unsuccessful occlusion of the left atrial appendage.

Furthermore, peripheral device leakage (PDL), or residual leak, remains a concern, particularly in combined procedures. Studies have suggested that residual leak after left atrial appendage closure (LAAC) may be associated with thromboembolism, increasing the risk of stroke [20]. While minimal residual leakage (< 1 mm) is generally considered less worrisome [21], the reliability of findings regarding the size of peripheral device leakage (PDL) and its impact on clinical outcomes remains debatable.

The National Cardiovascular Data Registry Left Atrial Appendage Occlusion (NCDR LAAO) registry indicated that patients with small PDL (< 5 mm) may be associated with a higher incidence of adverse clinical events after LAAC compared to those with large PDL (> 5 mm) or no PDL [22]. However, the asymmetry of cohort sizes and variations in antithrombotic treatments between groups raise questions about the reliability of these results. In our opinion, the enlargement of residual flow size is a more pertinent concern than the size itself.

Although the incidence of residual leak tends to increase in atrial fibrillation (AF) patients undergoing LAAC alone, the impact of catheter ablation (CA) on residual leak in the LAAC procedure remains uncertain. Most studies on combined LAAC with CA follow an ablation-first strategy. However, CA-induced left atrial appendage (LAA) oedema can lead to an underestimation of the LAA orifice, resulting in the implantation of a smaller device and incomplete occlusion with a residual flow exceeding 5 mm. Recent research suggests that combined procedures are associated with a higher incidence of residual leak, primarily attributed to CA. Hence, a device size recommendation of 20% or more larger than the LAA diameter, as opposed to the 10–20% often recommended, has been proposed for patients undergoing combined procedures [23]. Additionally, findings from the Western Atrial Fibrillation Ablation Strategy and Practice (WASP) registry underscore the need for larger occlusion devices in the Asian population due to their larger average LAA diameter [24]. In our study, the incidence of residual leak after the procedure did not significantly differ between the two groups, aligning with previously reported low rates. While our results do not fully support conclusions drawn by certain studies, we nonetheless recommend employing LAAC devices with larger sizes to minimize the risk of residual leak in patients undergoing combined procedures.

Antithrombotic therapy is commonly recommended to mitigate the risk of device-related thrombosis (DRT) and stroke in patients undergoing left atrial appendage closure (LAAC). However, balancing the need for anticoagulation with the risk of bleeding events remains a critical concern. Historically, the PROTECT AF study proposed an antithrombotic strategy involving 45 days of warfarin post-procedure, followed by 6 months of dual antiplatelet therapy (aspirin and clopidogrel) and subsequent lifelong aspirin use [25]. Nonetheless, the PREVAIL trial, employing a similar antithrombotic strategy, demonstrated a disparity in the rates of major bleeding events (0.4% in PREVAIL vs. 3.5% in PROTECT AF) [26].

Recent research suggests that shortening the duration of post-procedure anticoagulation may help reduce the incidence of bleeding events while maintaining efficacy in preventing DRT and stroke [27]. However, challenges such as variations in cohort populations and differences in medication adherence have limited the establishment of a universally accepted international consensus on post-LAAC antithrombotic strategies. Moreover, tailored anticoagulation strategies may be necessary for patients with unique clinical situations or those with different implanted devices. Achieving an optimal balance between preventing thromboembolic events and minimizing bleeding risks remains an ongoing area of investigation in the field of LAAC.

### Can combined procedure bring more long-term clinical benefits compared with LAAC alone?

The comparative evaluation of long-term clinical benefits between combined procedures (LAAC + PVI) and LAAC alone remains an active area of research. While LAAC has shown superiority to oral anticoagulants (OACs) in reducing the risk of stroke, evidence regarding the combined procedure is still limited.

In previous single-centre retrospective studies, Mo et al. found that the combined procedure had comparable efficacy to LAAC alone in preventing stroke [16], while Zhang et al. reported a significantly lower incidence of thrombotic events during follow-up in patients undergoing the combined procedure compared to LAAC alone [18].

In our study, the observed incidence of thrombotic events was 7.8% in the LAAC alone group and 4.0% in the combined procedure group. This rate appears relatively high when compared to findings from other studies. However, when assessed using person-years rate calculation, the thrombotic event rate was 2.6 per person-year in the LAAC alone group and 1.31 per person-year in the combined procedure group. Although this represents a significant decrease, it remains higher than the rates reported in the PROTECT AF trial [25] and a European study [28]. We attribute this difference to potential variations in race and geographical region among the study populations, as well as challenges related to poor follow-up compliance with anti-thrombotic medication. Notably, the reported incidence of ischemic infarction in Asia is higher than in Europe and the United States [29], and suboptimal adherence to follow-up protocols is a pervasive issue not only in our study but also nationally [30]. Additionally, we cannot entirely exclude the possibility that some patients had preexisting cerebrovascular conditions, contributing to the occurrence of strokes after LAAC.

No significant differences were observed in major adverse cerebrocardiovascular events (MACCE), rehospitalization due to cardiovascular disease (CVD), and rehospitalization due to atrial tachycardia (AT) between the combined procedure group and the LAAC alone group. This aligns with findings from the study by Mo et al. [16], where the incidence of stroke was comparable between the combined procedure and LAAC alone groups. However, it’s noteworthy that Zhang et al., in their study with a larger sample size, reported a significantly lower incidence of thrombotic events in the combined group compared to the LAAC alone group [18]. Differences in study populations, sample sizes, and follow-up durations could contribute to variations in outcomes across studies.

The relatively smaller sample size in our study compared to Zhang et al.’s study might be one reason for not finding significant differences in clinical follow-up events between the combined procedure and LAAC alone groups. Additionally, the possibility of patients undergoing repeat ablations during the follow-up period could impact the incidence of stroke and other cardiovascular adverse events, potentially narrowing the differences between the two groups.

In summary, the interpretation of clinical outcomes in combined procedures versus LAAC alone may be influenced by factors such as sample size, patient characteristics, and the follow-up duration. Future studies with larger cohorts and rigorous study designs will contribute to a more comprehensive understanding of the comparative effectiveness of these procedures.

Regarding the AF recurrence rate, we conducted a survival analysis comparing patients who underwent the combined procedure with those who underwent PVI alone, as illustrated in Fig. 1f. The results revealed that patients who underwent PVI alone had an AF recurrence rate of 15.8% over a mean follow-up of 26.7 ± 7.9 months. This rate was lower than that observed in patients who underwent the combined procedure (28%), but the difference between the two groups was not statistically significant (Log-Rank *p* = 0.075). This finding aligns with a randomized study reported by Romanov et al. [13]. However, further research necessitates randomized, prospective studies for more conclusive insights.

In a recently published study investigating the impact of the combined procedure on left atrial function, Yang et al. demonstrated that the combined procedure can improve left atrial ejection function during long-term follow-up. Notably, the beneficial effect primarily arises from ablation rather than LAAC [31]. Similarly, Wang et al. showed a significant improvement in left ventricular ejection fraction (LVEF) in AF patients undergoing either the combined procedure or catheter ablation (CA) alone. In contrast, patients undergoing drug therapy alone or LAAC alone experienced a decrease in LVEF after a 1-year follow-up [32]. Our study aligns with these findings, indicating that the average LVEF of patients in the combined group without AF recurrence exhibited significant improvement during post-procedure follow-up. In contrast, there was no significant change in average LVEF in the LAAC alone group and the combined group with AF recurrence. This suggests that heart function can benefit from the control of heart rhythm in patients undergoing the combined procedure. We postulate that the improvement in heart function may be attributed to patients undergoing the combined procedure achieving sinus rhythm through CA, thereby achieving a better hemodynamic status in the left atrium (LA). We believe that the enhancement of LA function by restoring sinus rhythm, allowing blood to fully fill and eject from the left atrium, contributes to the increase in LVEF in patients undergoing the combined procedure during follow-up. Therefore, we contend that the increase in LVEF not only reflects the improvement of left ventricular (LV) function but also indirectly signifies the improvement of LA function. In contrast, the role of LAAC in improving cardiac function remains controversial. Some small-scale studies have reported the occurrence of acute heart failure (AHF) after LAAC, mostly attributed to preexisting heart failure [33–35]. However, as a part of the left atrium, the left atrial appendage (LAA) is partially responsible for accommodating blood volume and pressure in the left atrium. It regulates hemodynamic through the secretion of natriuretic peptides [36].

Bartus et al. demonstrated, for the first time, that epicardial left atrial appendage occlusion (LAAC) using the LARIAT device has a long-term effect on lipid and glucose metabolism [37]. Additionally, they observed a reduction in systolic and diastolic blood pressure at 1 year and 2 years after epicardial LAAC, indicating a systemic effect resulting from left atrial appendage occlusion and its impact on endocrine function [38]. Upon completion of left atrial appendage (LAA) occlusion, there is an acute reduction in the volume of the total left atrial (LA) chamber, accompanied by an increase in LA pressure, leading to the inhibition of LAA endocrine function [39, 40]. These factors can contribute to the development of post-left atrial appendage occlusion acute heart failure (AHF).

However, with the progress of ablation technology and the emergence of new ablation strategies, such as LAA ligation, which can achieve both complete electrical isolation of the LAA and LAA occlusion, we believe that patients can benefit more from a combined procedure.

## Limitations

Our study is a retrospective single-centre propensity score-matched (PSM) study with a limited study population, thereby limiting the generalizability of the conclusions. The specific inclusion criteria restrict the application of our findings to general atrial fibrillation (AF) patients. While PSM was employed to mitigate differences in baseline characteristics, it simultaneously reduced the overall study population size. Moreover, deviations from recommended instructions for the timing and frequency of transoesophageal echocardiography (TOE) examinations may have led to an underestimation of device-related complications. It is important to note that not all patients included were primary AF patients, and both groups comprised individuals who had undergone pulmonary vein isolation (PVI) or other ablation procedures previously, potentially impacting the study’s conclusions. Consequently, there is a need for further randomized, long-term, large-scale, prospective clinical studies on this subject.

## Conclusion

In our propensity score-matched study, the addition of pulmonary vein isolation (PVI) to left atrial appendage closure (LAAC) did not result in an increase in procedure-related complications compared to LAAC alone. However, the combined procedure did not confer significant advantages to atrial fibrillation (AF) patients in terms of preventing thrombotic events, major adverse cerebrocardiovascular events (MACCE), or re-hospitalization due to cardiovascular disease (CVD) and atrial tachycardia (AT). Nonetheless, combined LAAC with ablation for atrial fibrillation could be the most comfortable way for AF patients to achieve both thromboembolism protection by LAAC and heart function improvement by atrial fibrillation ablation.

### Supplementary Information

**Additional file 1: Supplemental table 1. **Baseline characteristics of PVI alone groups versus combined group.

## Data Availability

The datasets used and analysed during the current study are available from the corresponding author upon reasonable request.

## Abbreviations

AAD: Anti arrhythmic drugs
ACEI/ARB: Angiotensin-converting enzyme inhibitor/angiotensin II receptor blocker
AHF: Acute heart failure
AF: Atrial fibrillation
AT: Atrial tachycardia
BMI: Body mass index
CA: Catheter ablation
COPD: Chronic obstructive pulmonary disease
CVD: Cardiovascular disease
DAPT: Double antiplatelet
eGFR: Estimated glomerular filtration rate
LAAC: Left atrial appendage closure
LAD: Left atrial diameter
LVEF: Left ventricular ejection fraction
MACCE: Major adverse of cerebral and cardiovascular events
NOACs: New oral anticoagulants
OAC: Oral anticoagulants
pro BNP: Pro-brain natriuretic peptide
PSM: Propensity score matching
PVI: Pulmonary vein isolation
RF: Residual flow
SAPT: Single antiplatelet
TOE: Transoesophageal echocardiography
TIA: Transient ischemic attack
TTE: Transthoracic echocardiography
