# Supplementary material for: Long-term outcomes of left atrial appendage closure with or without concomitant pulmonary vein isolation:a propensity score matching analysis based on CLACBAC study
Source: BMC Cardiovasc Disord. 2024 Feb 3;24:85. doi: 10.1186/s12872-024-03725-1 (PMC10837861; doi:10.1186/s12872-024-03725-1)
Supplement: Supplementary file 1 — Additional file 1: Supplemental table 1. Baseline characteristics of PVI alone groups versus combined group. [file 12872_2024_3725_MOESM1_ESM.docx]

**Supplemental table 1.** Baseline characteristics of PVI alone groups versus combined group.

| **Baseline** | **Combined procedure (n=57)** | **PVI alone (n=57)** | **P value** |
| --- | --- | --- | --- |
| Age, years | 71.4±8.1 | 71.6±7.0 | 0.991 |
| Men, n (%) | 26 (45.6) | 32 (56.1) | 0.349 |
| AF type |  |  | 0.336 |
| Paroxysmal, n (%) | 32 (56.1) | 38 (66.7) |  |
| Persistent, n (%) | 25 (43.9) | 19 (33.3) |  |
| CHA_2_DS_2_-VAS_C_ score | 4.0 [2.0,5.0] | 3.0 [2.0,4.0] | <0.005* |
| HAS-BLED score | 2.0 [2.0,3.0] | 2.0 [2.0,2.0] | 0.162 |
| LAD, mm | 41.0 [36.0,44.0] | 41.0 [37.0,44.0] | 0.860 |
| LVEF, % | 60.0 [58.0,62.0] | 60.0 [58.0,63.0] | 0.418 |

AF, atrial fibrillation; LAD, left atrial diameter; LVEF, left atrial ejection fraction; PVI, pulmonary vein isolation.

*, significant difference
